# Supplementary material for: Comparing intramuscular adipose tissue on T1-weighted and two-point Dixon images
Source: PLoS One. 2020 Apr 9;15(4):e0231156. doi: 10.1371/journal.pone.0231156 (PMC7144956; doi:10.1371/journal.pone.0231156)
Supplement: Attachment — Submitted filename: Response to Reviewers.docx [file pone.0231156.s001.docx]

Dear Dr. Sanada,

Thank you for inviting us to submit a revised draft of our manuscript entitled, “Comparing intramuscular adipose tissue on T1-weighted and two-point Dixon images” to *PLOS ONE*. We also appreciate the time and effort you and each of the reviewers have dedicated to providing insightful feedback on ways to strengthen our paper. Thus, it is with great pleasure that we resubmit our article for further consideration. We have incorporated changes that reflect the detailed suggestions you have graciously provided. We also hope that our edits and the responses we provide below satisfactorily address all of the issues and concerns that you and the reviewers have noted.

To facilitate your review of our revisions, the following is a point-by-point response to the questions and comments delivered in your letter dated November 15, 2019.

**Response to Reviewer #1**

Thank you very much for providing important insights. In the following sections, you will find our responses to each of your points and suggestions. We are grateful for the time and energy you expended on our behalf.

**Comment Q1:** *The authors concluded that T1W imaging is likely to be the better option for	IntraMAT measurements. It is ambiguous about the process of this conclusion. Therefore, the	authors need to discuss the more clearly. IntraMAT content determined by T1W was significantly higher than that determined by 2PD in the AM and BF-L. Which value is appropriate?*

**Response A1**: We agree that this point requires clarification. The gold standard for IntraMAT measurement is muscle biopsy, but MRI is more suitable for IntraMAT than CT (Mitra et al. 2017). However, it is not clear from previous studies which MR imaging method is better. T1-weighted imaging (T1W) can measure IntraMAT content even with a low magnetic field MR scanner, and has been used in many previous studies. However, the adipose tissue and muscle segmentation technique was different between research teams (Ogawa et al. 2017). On the other hand, the Dixon method is recommended for MRI with a 1.5T or higher because of its technical limitations in being able to clearly distinguish between water and fat signals. We indicated in the original manuscript that T1W is better suited for measuring IntraMAT content than the Dixon method in clinical scenarios, because of the characteristics of each sequence and our study’s results showing no significant difference in IntraMAT content between T1W and two-point Dixon imaging for the vastus lateralis. However, we did not obtain muscle biopsies in this study, and could not conclude which value of T1W or Dixon was appropriate. Therefore, we changed the following text in the DISCUSSION (page 20, lines 7-10):

“IntraMAT content by both T1W and 2PD methods was consistent or correlated with muscle biopsy in previous studies. However, when T1W imaging is used, extra care must be taken of the IntraMAT content discrepancy with 2PD found in this study. (page 20, lines 7-10)”

We deleted the next part in the CONCLUSION (page 21, line 13):

“T1W imaging is likely to be the better option for IntraMAT measurements, taking into consideration the potential discrepancies that can arise when calculating IntraMAT values.”

**Comment Q2:** *What is the gold standard to assess IntraMAT? T1W? Dixon? biopsies?*

*Please describe in the introduction.*

**Response A2:** The gold standard for IntraMAT measurement is muscle biopsy; we added the following text to indicate this (page 5, line 2 to 4):

“Previous studies confirmed that IntraMAT content by both the T1W and Dixon methods were consistent or correlated with muscle biopsy, which is the gold standard to measure IntraMAT [20,21].”

**Comment Q3:** *Is the order of measurement randomized? Can the authors clarify the study protocols?*

**Response A3:** We set the MRI sequence from T1W to 2PD imaging in a sequential order. MR imaging was performed by a radiological technologist who was not interested in this study, so we could not change the MR imaging order. MR images were read in random order for analysis. However, we could not clarify the study protocols. Therefore, we compared the value between randomization order and original order analysis on 10 subjects (TABLE). We used block randomization (Kim and Shin, 2014). IntraMAT content was not different between randomization order and original order (*P* = 0.218 to 0.896) in all muscles. We added the following text (page 8, line 15):

“MR images were read in random order for the analysis.”

TABLE. Effect of analysis order in intramuscular adipose tissue content (IntraMAT content; %) in T1W and 2PD Images (N=10).

|  | Mean ± SD (%) | |
| --- | --- | --- |
|  | Original analysis order | Randomization order |
| Vastus lateralis  T1W  2PD | 11.7 ± 5.5  12.7 ± 3.1 | 11.3 ± 6.6  13.4 ± 3.9 |
| Adductor magnus  T1W  2PD | 11.3 ± 5.7  8.6 ± 1.7 | 12.3 ± 5.0  8.6 ± 1.9 |
| Biceps femoris-long head  T1W  2PD | 21.7 ± 12.9  13.8 ± 4.5 | 19.9 ± 16.5  13.4 ± 3.9 |

Abbreviations: T1W, T1-weighted; 2PD, 2-point Dixon

**Comment Q4:** *In the Table 1, the authors should present the comparison between young and older. It will be interesting to know whether the effects of aging differ between T1W and 2PD methods.*

**Response A4:** We investigated the effects of aging differences between the T1W and 2PD methods. We included statistical results in Table 1 (page 12, line 7 to 11) to further illustrate the statistical analysis and RESULTS. We added the following texts (page 11, line 10 and 11, page 12 line 5 to 6): “We compared IntraMAT content by the two MRI methods between the younger and the older men using Student’s unpaired *t*-test.”

“In the older group, IntraMAT content was significantly higher than in the younger group in all muscles and with both the T1W and 2PD methods (Table 1).”

**Comment Q5:** *Instead of showing only the relationship between difference in IntraMAT contents and difference in signal intensity, Figure should present the relationship between IntraMAT content determined by T1W and 2PD.*

**Response A5：**We have included a new Fig 2 to further illustrate this in the RESULTS. We added the following Fig 2 and associated texts (page 13, line 1 to 7).

“Fig 2. Relationship between the intramuscular adipose tissue content (IntraMAT content; %) using T1-weighted (T1W) and 2-point Dixon (2PD) images in the vastus lateralis (VL), adductor magnus (AM), and the biceps femoris-long head (BF-L).”

“The relationship between IntraMAT content determined using T1W and 2PD is shown in Fig 2. There was a high positive correlation between these two values using T1W and 2PD for the different muscles (VL; r = 0.735, *P* < 0.01, AM; r = -0.717, *P* < 0.01, BF-L; r = 0.790, *P* < 0.01, VL + AM + BF-L; r = 0.686, *P* < 0.01; Fig 2).”

**Comment Q6：***In the Figure 3(a new Fig 4), is there the representative image of Lower IntraMAT?*

**Response A6：**We apologize for this oversight. We inserted the image of Lower IntraMAT in Fig 4 (the figure below).

**Comment Q7：** *The authors described “the IntraMAT content of this study by 2PD which was three times higher than that found in previous studies”. How is T1W compared to previous studies? Please discuss.*
**Response A7：** In accordance with the reviewer's comment, we have added discussion about *the IntraMAT content of this study by* T1W (page 17, line 14 to 18). We added the following texts: “In the present study, the IntraMAT content by T1W was two times higher than that found in previous studies [13-15]. A previous study reported that IntraMAT contents of the VL were 3.6% and 7.7% in younger and older subjects, respectively, who were men and women [14]. Subject characteristics, physical activity levels, and sex differences could all explain these differences in IntraMAT values.”

**Comment Q8**. *Please change “positive correlation” to “negative correlation” (page 14, line 7).*

**Response A8**. This error has been corrected in accordance with the reviewer's comment (page 15, line 8).

**Response to Reviewer #2**

Thank you very much for providing important insights. In the following sections, you will find our responses to each of your points and suggestions. We are grateful for the time and energy you expended on our behalf.

**Comment 1:** *The MR image shows a change in contrast when there is a distance change from the magnetic field center in the gantry. Therefore, in addition to this experimental data, 1) Change the subject's imaging position, 2) After imaging at a site away from the center of the bore, recalculate to see if similar results are obtained.*

**Response 1:** We appreciate the reviewer's interest in additional information in addition to this experimental data. However, the purpose of our study was to investigate the difference in imaging methods at the same position. In this study, T1W and 2PD were taken continuously without moving the subject (page 7, line 12 to 15). Therefore, the subject's positioning and distance from the bore were constant for the two imaging methods. We consider that additional experimental data may not be necessary, and therefore wish to retain the original text.

**Comment 2:** *In addition, the calculated MR signal intensity depends on the voxel size. Therefore, change the Voxel size and verify that similar results are obtained.*

**Response 2:** We agree that additional information on voxel size as the reviewer suggested would be valuable. Regrettably, we used the MRI scanner (MAGNETOM Verio, Siemens Healthcare Diagnostics K.K., Tokyo, Japan) that has a limitation of being unable to match the voxel size using the two MR imaging methods. We confirmed this when setting up the T1W and 2PD sequences. Therefore, two MR images were taken with the closest voxel size in this study.

**Figures：**

To improve the manuscript according to the reviewer’s comments, we have changed the figure composition as described below:

The previous Fig 2 has been changed to Fig 3.

The previous Fig 3 has been changed to Fig 4.

The previous Fig 4 has been changed to Fig 5.

The previous Fig 5 has been changed to Fig 6.

A new Fig 2 has been inserted into the manuscript.

**REFERENCES**

Kim J, Shin W. (2014). How to do random allocation (randomization). Clin Orthop Surg, 6(1);103-109. doi:10.4055/cios.2014.6.1.103

Mitra S, Fernandez-Del-Valle M, Hill JE. (2017). The role of MRI in understanding the underlying mechanisms in obesity associated diseases. Biochim Biophys Acta Mol Basis Dis, 1863(5); 1115-1131. doi:10.1016/j.bbadis.2016.09.008

Ogawa M, Lester R, Akima H, Gorgey AS. (2017). Quantification of intermuscular and intramuscular adipose tissue using magnetic resonance imaging after neurodegenerative disorders. Neural Regen Res.12(12); 2100-2105. doi:10.4103/1673-5374.221170

Again, thank you for giving us the opportunity to strengthen our manuscript with your valuable comments and queries. We have worked hard to incorporate your feedback and hope you will agree that that these revisions have made our manuscript suitable for publication in *PLOS ONE*.

Sincerely,

Madoka Ogawa
Assistant Professor

Nippon Sport Science University

Address: 7-1-1 Fukasawa, Setagaya-ku, Tokyo 158-8508, Japan

E-mail address: [d0c0dem0.mad020@gmail.com](mailto:ogawa-ma@nagoya-u.jp)

Phone: +81-3-5706-0992
