# Supplementary material for: Comparing intramuscular adipose tissue on T1-weighted and two-point Dixon images
Source: PLoS One. 2020 Apr 9;15(4):e0231156. doi: 10.1371/journal.pone.0231156 (PMC7144956; doi:10.1371/journal.pone.0231156)
Supplement: Attachment — Submitted filename: Response to Reviewers0310.docx [file pone.0231156.s002.docx]

Dear Dr. Sanada,

Thank you for inviting us to submit a revised draft of our manuscript titled, “Comparing intramuscular adipose tissue on T1-weighted and two-point Dixon images” to *PLOS ONE*. We also appreciate the time and effort you and each of the reviewers have dedicated to providing insightful feedback on ways to strengthen our paper. Thus, it is with great pleasure that we resubmit our article for further consideration. We have incorporated changes that reflect the detailed suggestions you have graciously provided. We also hope that our edits and the responses we provide below satisfactorily address all of the issues and concerns that you and the reviewers noted.

To facilitate your review of our revisions, the following is a point-by-point response to the questions and comments delivered in your letter dated January 29, 2020.

**Response to Reviewer #1**

Thank you very much for your cooperation.

**Response to Reviewer #2**

We wish to express our deep appreciation to the reviewer for the insightful comment on this point. We apologize for not answering your previous question perfectly.

**Comment 1:** *It can be inferred from the text that T1w and the 2PD method was performed continuously. Since it is described as "consective," it is not necessary to describe it as "continuously."*

**Response 1:** Thank you for your suggestion. We deleted “continuously” (page 7, line 14).

**Comment 2:** *As you know and use it, MR devices have different image data and contrast (power from changes in SN and* *SAR) depending on continuous imaging and shimming conditions. These problems have been corrected in the image conversion process. If it is far from the center of the gantry, the effect of local distortion, due to MR, also appears. It is not a matter of continuity, but the MR system. In addition, the distance between the body-coil and the measurement site is also an issue.
This paper is a valuable paper that utilizes an uncomplicated evaluation and hopes for future development. Therefore, it is essential that the reliability of the published data is established and whether the reader can obtain comparative data by the same method. I understand your response is giving up its verification. The previous advice does not consider continuous imaging as a problem but points out the reproducibility of data. I highly recommend you describe problems with MR equipment properly. The advice for voxel size is the same. These points should also be considered in the limitations. If you use a method that can be evaluated easily, it is recommended that you consider it properly.*

**Response 2:** We agree with your suggestion and added SNR results between T1W and water, and T1W and fat images by 2PD. In addition, we added SNR-related discussion and Figure 7a and b. Also, we added the limitation of our inability to use the same voxel size between the T1W and 2PD imaging methods in this study. We added the following text (page 20, line 2 to 18), Figure 7a and b (page 21, line 1) and reference (page 29, line 15 to 18).

In addition, we considered that the location of muscles and imaging methods could be related changes in the signal-to-noise ratio (SNR). We measured SNR with the subtraction method, which is the most commonly used method [35]. In this method, a difference image was obtained by subtracting two identical images. We measured the SNR between T1W and water images, and T1W and fat images for 10 subjects, in the same size ROI of four corners (ROI 1 to ROI 4) to compare the SNR between the ROIs. The SNR in ROI 4 was a significantly higher than in ROI 1, but there was no significant difference between the other ROIs (Fig.7a and b). In this study, VL was on the ROI 3 side, AM was on the ROI 2 side, and BF-L was on the ROI 4 side. These results suggest that the effect of SNR was not observed independent of the location of muscles in this study. Unfortunately, we could not use the same voxel size between the T1W and 2PD imaging methods because of the MR device; therefore, the effect of the SNR on the results of IntraMAT could not be excluded. Furthermore, the effect of local distortion in the MR by distance of the center of the gantry to the body coil and the measurement site could also be related to the difference in IntraMAT content between T1W and 2PD. Thus, this study had some important limitations. Further human and phantom experiments under stable conditions of voxel size, SNR, and specific absorption rate would be required to reveal what caused the difference in IntraMAT content between T1W and 2PD.

**Fig 7. Difference of four corners in the signal-to-noise ratio (SNR) between T1-weighted (T1W) and water images, and T1W and fat images.**

^*^*P* < 0.05, vs. regions of interest (ROI) 1

**REFERENCE**

35.	Ohno N, Miyati T, Niwa Y, Kan H, Ishida S, Kasai H, et al. Novel practical SNR determination method for MRI using double echo with longest second echo time (DELSET). Br J Radiol. 2018;91(1086):20170652. Epub 2018/03/23. doi: 10.1259/bjr.20170652. PubMed PMID: 29565674; PubMed Central PMCID: PMCPMC6223277.

Again, thank you for giving us the opportunity to strengthen our manuscript with your valuable comments and queries. We have worked hard to incorporate your feedback and hope you will agree that that these revisions have made our manuscript suitable for publication in *PLOS ONE*.

Sincerely,

Madoka Ogawa
Assistant Professor

Nippon Sport Science University

Address: 7-1-1 Fukasawa, Setagaya-ku, Tokyo 158-8508, Japan

E-mail address: [d0c0dem0.mad020@gmail.com](mailto:ogawa-ma@nagoya-u.jp)

Phone: +81-3-5706-0992
